# Resolving sequential electron–proton transfer kinetics for electrochemical CO_2_ reduction at the Cu(100)/H_2_O interface *via* a quantum-classical framework

**DOI:** 10.1039/d5sc07385e

**Published:** 2026-02-02

**Authors:** Yun Yang, Gang Fu

**Affiliations:** a College of Chemistry and Chemical Engineering, Xiamen University Xiamen China gfu@xmu.edu.cn +86-592-2183047 +86 13625012808

## Abstract

Electron-transfer (ET) and proton-transfer (PT) events occurring at electrochemical interfaces ultimately dictate the efficiency of electrocatalytic energy conversion and chemical synthesis. Despite their significance, a unified theoretical description of ET/PT dynamics has been challenging due to the entangled electronic, nuclear, and solvent degrees of freedom. Herein, we present a quantum-classical multiscale framework integrating constrained density functional theory (CDFT) with machine learning accelerated molecular dynamics (MLMD) to investigate ET and PT during electrochemical CO_2_ reduction on Cu(100) in explicit water. Distinct ML potentials are trained for the adiabatic ground state and for two charge-localized diabatic states, enabling efficient configurational sampling while preserving quantum-mechanical fidelity in electronic energies and forces. The derived diabatic free-energy surfaces reveal that CO_2_ first undergoes inner-sphere ET to yield chemisorbed *CO_2_^−^, followed by PT to form *COOH. Solvent reorganization imposes kinetic constraints on the ET step yet counterintuitively stabilizes the *CO_2_^−^ intermediate through ion–dipole interactions, modulating the vibronic couplings. For PT, solvent relaxation dynamically adjusts the equilibrium donor–acceptor distance, thereby augmenting the Franck–Condon overlap between reactant and product vibronic wavefunctions in excited proton vibrational states, which facilitates nonadiabatic transitions across diabatic surfaces. Rate constants extracted by combining diabatic vibronic PCET theory with generalized Langevin equation-derived Grote–Hynes theory show that the sequential ET–PT pathway outpaces concerted PCET by about 5 orders of magnitude at the potential of zero charge (PZC). This methodology establishes a robust paradigm for dissecting ET and PT kinetics at electrochemical interfaces, emphasizing the interplay of quantum nuclear effects, vibronic couplings, and solvent fluctuations.

## Abstract 2

Multiscale simulations integrating machine learning and diabatic dynamics uncover the sequential electron transfer–proton transfer pathways in CO_2_ electroreduction, highlighting the role of solvent fluctuations and quantum nuclear effects.

## Graphical abstract

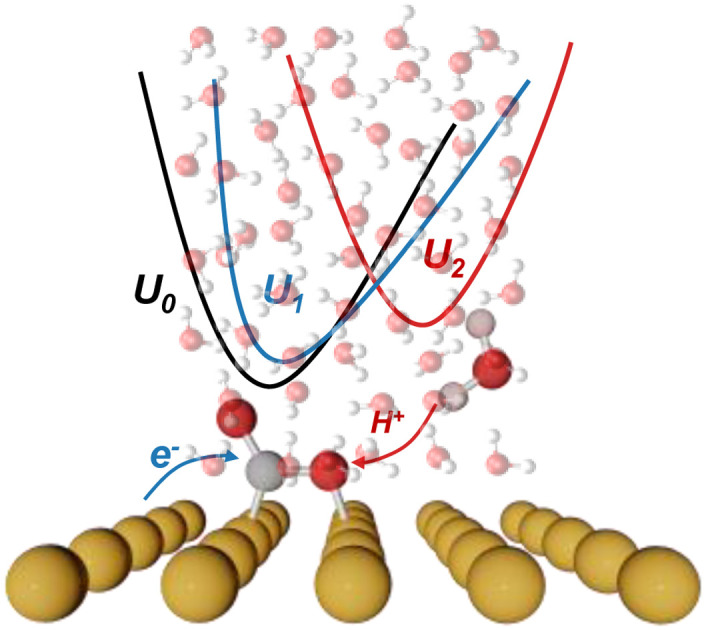

## 1 Introduction

The electrochemical reduction of CO_2_ (CO_2_RR) represents a compelling strategy for sustainably producing fuels and commodity chemicals, because it couples renewable electricity with CO_2_ valorization and thus mitigates anthropogenic emissions.^1–5^ Maximizing catalytic performance hinges on unequivocally identifying the rate-determining elementary step, which consensus attributes to coupled electron-transfer (ET) and proton-transfer (PT) events at the electrode–electrolyte interface.^6–9^ Whether these transfers proceed sequentially *via* an inner-sphere ET followed by PT, or occur in a single concerted proton-coupled electron transfer (PCET) step, however, remains unresolved.^10–12^ Conventional periodic density functional theory (DFT) calculations often favor a concerted PCET pathway for aqueous CO_2_RR on metal/H_2_O interfaces, whereas recent electro-kinetic studies point to a rate-limiting one-electron step whose kinetics are strongly modulated by the interfacial solvent structure.^13,14^ This apparent discrepancy underscores the need for developing a theoretical framework that treats electrons, protons, and the dynamic solvent on an equal footing.

Several methodological limitations hinder such a framework. First, standard Kohn–Sham DFT is intrinsically adiabatic and cannot describe the nonadiabatic ET and PT processes that typify electrochemical reactions.^15–18^ Constrained DFT (CDFT) can generate the requisite diabatic potential energy surfaces (PESs), yet exhaustive sampling of condensed-phase interfaces with CDFT remains computationally prohibitive. Second, thermodynamic integration (TI) proceeds along an alchemical coupling parameter rather than explicit collective variables, and thus ignores the stochastic coupling between the adsorbates, the polarizable metal surface, and the fluctuating hydrogen-bond network.^19–21^ Third, kinetic models based on the Newns–Anderson Hamiltonian and Fermi's golden rule assume weak electronic coupling, an assumption that breaks down for chemisorbed intermediates that hybridize strongly with the Cu d-band.^22^ Finally, nuclear quantum effects (NQEs) – most notably proton tunnelling and zero-point motion can decisively influence kinetics but are routinely neglected in continuum or classical-MD solvent models.^23,24^ Rectifying these deficiencies demands: (i) accurate diabatic landscapes that explicitly localize electronic charge; (ii) explicit, dynamical solvent models that capture reorganization energetics; and (iii) a quantum-mechanical treatment of proton motion to include tunnelling and kinetic isotope effects.

In this contribution, we deploy an integrated framework fusing CDFT with machine learning potentials (MLPs) to expedite molecular dynamics at the Cu(100)/H_2_O interface. Distinct MLPs, trained for the adiabatic ground state and two charge-localized diabatic PESs, facilitate comprehensive configurational sampling while upholding quantum accuracy. Simulations reveal a prevailing sequential ET–PT mechanism: inner-sphere ET yields adsorbed *CO_2_^−^, succeeded by PT to *COOH. A direct PCET pathway from physisorbed CO_2_ is kinetically disfavored by a substantially higher barrier. Moreover, frequency-resolved analysis of the solvent friction kernel at the diabatic crossing uncovers specific interfacial vibrational modes that couple strongly to the reaction coordinate and dissipate energy. These findings demonstrate the necessity of multiscale, quantum-classical simulations that integrate electronic, nuclear, and solvent degrees of freedom to achieve a mechanistic understanding of electrochemical PCET processes.

## 2 Computational methods

### 2.1 Electronic structure

CDFT emerges as a robust methodology for extracting electron transfer (ET) parameters in condensed-phase systems, surpassing perturbative approaches by ensuring superior asymptotic behavior, global smoothness of diabatic potentials, and computational tractability.^25^ In this work, CDFT-based molecular dynamics simulations of CO_2_ at the Cu(100)/H_2_O interface were executed within the CP2K software package.^26^ The interface model comprised a 5 × 5 supercell with five Cu layers, described using the PBE-D3(BJ) functional.^27,28^ The slabs were segregated by approximately 32 Å of explicit H_2_O containing 170 molecules, maintaining an interfacial water density of about 1 ± 0.05 g cm^−3^ far from the surfaces.

### 2.2 Molecular dynamics

To efficiently explore configurational space and accurately capture interfacial energetics, deep neural networks (DNNs) were trained using energies and forces derived from CDFT calculations,^29,30^ facilitated by a concurrent learning scheme within the ai2-kit toolkit.^31,32^ Three separate MLPs were generated: one representing the adiabatic ground state and two corresponding to diabatic states localized with net charges of 0|*e*| and −1|*e*| on the CO_2_, respectively. These charge-localized states were rigorously defined by optimizing constraint potentials using Lagrange multipliers within modified Kohn–Sham equations. The resulting MLPs effectively reproduced both CDFT and conventional DFT energies, ensuring accurate construction of diabatic and adiabatic free-energy surfaces (FESs). The reliability of these MLPs is demonstrated by their excellent performance on both training and test datasets, with detailed accuracy metrics provided in Fig. S2–S7. MD simulations employing these MLPs were carried out using the LAMMPS and PLUMED packages.^33,34^ Simulations were conducted within the canonical ensemble (NVT), maintained at 330 K using a Nosé–Hoover thermostat, and integrated with a 0.5 fs timestep. Helmholtz free energies were calculated through well-tempered metadynamics (WT-MetaD), with Gaussian bias and collective variable parameters thoroughly detailed in the SI.

### 2.3 Rate theory

Accurate determination of PCET rate constants hinges upon the magnitude of vibronic coupling connecting reactant (*µ*) and product (*ν*) electron–proton vibronic states. These couplings are quantified by Hamiltonian matrix elements:^35^

1 *V*_*aµkν*_ = *V*_*ak*_*S*_*µν*_

here *V*_*ak*_ is the electron coupling between reactant electronic state |*a*〉 and delocalized wavefunctions |*k*〉 of the quasi-free electrons in the metal, while *S*_*µν*_ represents the vibrational overlap integral between reactant and product proton vibrational states, *i.e.* 〈*χµ*^*a*^*|χ*_*ν*_^*k*^〉. In the vibronically adiabatic regime, the PCET rate constant is accurately approximated by transition state theory (TST) modified by Grote–Hynes corrections^36,37^ yielding:2
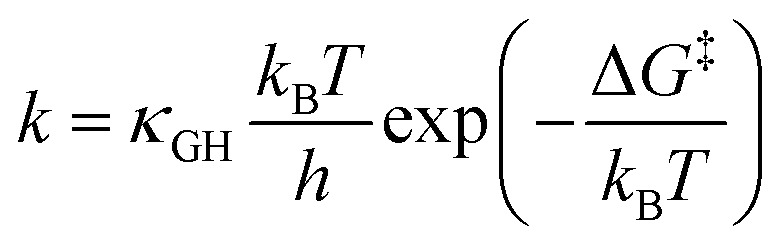
here, Δ*G*^‡^ represents the free-energy barrier, *k*_B_ is the Boltzmann constant, *T* is the temperature, *h* is Planck's constant, and *κ*_GH_ is the Grote–Hynes transmission coefficient. However, within the diabatic vibronic formalism, the PCET rate explicitly depends on the vibronic coupling. For ET processes, the nonadiabatic rate constant can be expressed as:^38^3
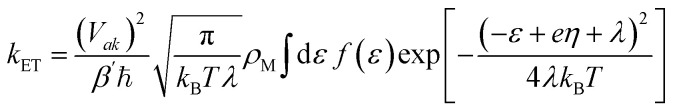


For fixed proton donor-accept distance *R*, the Landau–Zener nonadiabatic PCET rate constants are evaluated as:^39–41^4
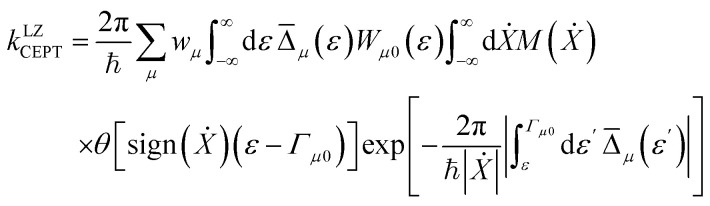
where *w*_*µ*_ is the Boltzmann probabilities for the proton vibrational state *µ*; 
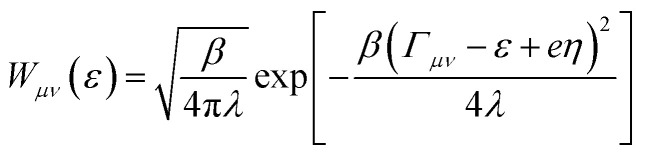
 is the probability of sampling a crossing point at 
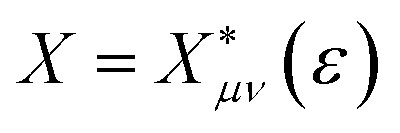
. 
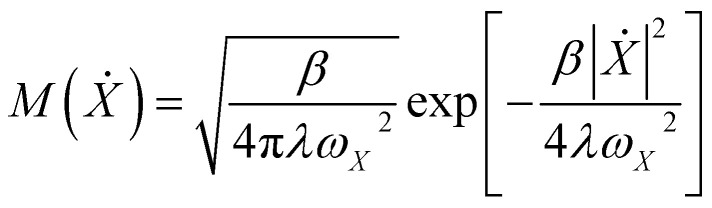
 is the Maxwell distribution of velocities *Ẋ*, *ω*_*X*_ is the harmonic frequency along the collective reaction coordinate *X*, *θ* is the Heaviside step function, and 
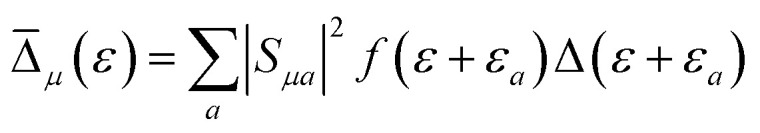
 is the cumulative weighted density of states for the occupied levels of the electrode. *f*(*ε*) is the Fermi–Dirac distribution function for the electronic states in the metal, *ρ*_M_ is the density of states at the Fermi level, *β*′ is a parameter representing the exponential decay of the electronic coupling with the distance between the molecule and the electrode, *λ* is the reorganization energy, and *η* is the applied potential. Notably, weak electronic coupling with macroscopic electrodes justifies the electronically nonadiabatic approximation per vibronic pair, permitting a truncated Landau–Zener expansion for transitions. Yet, the quasi-continuum of product states may confer apparent adiabaticity to the ensemble rate. Proton donor–acceptor motion is incorporated *via* thermal averaging:^42^

5 *k*_tot_ = ∫d*R* *P*(*R*)*k*_CEPT_(*R*)

where *P*(*R*) are probability distribution functions for *k*_CEPT_(*R*).

## 3 Results and discussions

### 3.1 Free-energy landscapes

The adiabatic and diabatic state free-energy landscapes of inner-sphere ET and CEPT are extracted from WT-MetaD simulations based on the MLPs. The primary reaction coordinate for inner-sphere ET is defined as the centroid distance along the *z*-axis between CO_2_ and the nearest Cu(100) surface atom. For the subsequent protonation of *CO_2_ to *COOH, two collective variables, *R*_1_ and *R*_2_ (illustrated in Fig. S1), are utilized to capture the proton transfer dynamics.

**Fig. 1:**
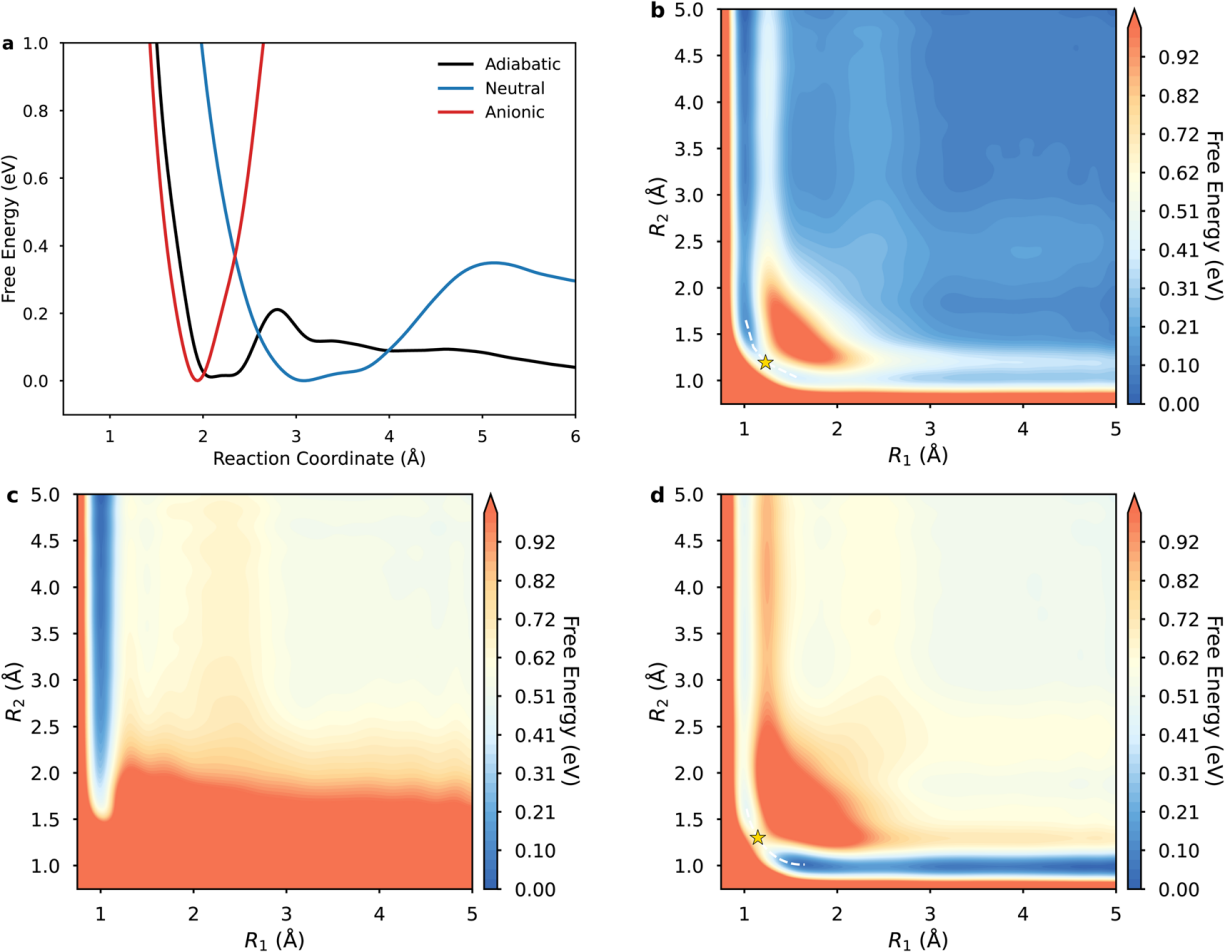
Diabatic free-energy surfaces for CO_2_ reduction on Cu(100). (a) One-dimensional profile for the inner-sphere ET step, plotted along the CO_2_-surface centroid distance. (b–d) Two-dimensional free-energy landscapes for PCET projected onto the collective variables *R*_1_ and *R*_2_ on (b) the adiabatic ground state, (c) the neutral diabatic surface, and (d) the anionic diabatic surfaces.

As shown in Fig. 1a, the FESs of adiabatic and diabatic state 2 (anionic CO_2_^−^) exhibit nearly coincident minima in the product region. In contrast, in the reactant regime beyond a reaction coordinate of about 5 Å, the adiabatic surface aligns with diabatic state 1 (neutral CO_2_). The essential distinction arises in the intermediate region, where the diabatic states intersect to form a crossing seam that mediates nonadiabatic transitions between electronic states. It is important to emphasize, however, that adiabatic and diabatic states are defined on distinct theoretical energy scales; therefore, direct comparison of absolute energies between the two is not physically meaningful. Crucially, the diabatic FESs intersect at 2.42 Å with a barrier of 0.37 eV, defining a nonadiabatic seam critical to interfacial ET dynamics. The displacement between the diabatic crossing (2.42 Å) and the adiabatic barrier (2.75 Å) reflects the additional free-energy cost associated with solvent reorganization required to stabilize the *CO_2_^−^ species after the initial electronic interaction becomes favorable. Consistently, the net charge of CO_2_ (Fig. S8) along the reaction coordinate also transition near the adiabatic barrier maximum. Following chemisorption and concurrent ET completion, PT from H_2_O yields *COOH. The adiabatic ET–PT exhibits a reaction free energy of 0.27 eV and barrier of 0.34 eV. On the neutral diabatic surface-permitting only concerted PCET, the FES rises monotonically from reactants to products, devoid of a well-defined transition state and with a reaction free energy surpassing 1 eV, signifying that *COOH formation demands prior electron injection into CO_2_. Conversely, the post-ET anionic diabatic surface displays *CO_2_^−^ conversion to *COOH with a moderate barrier of 0.19 eV and free energy of −0.44 eV. Together, these observations substantiate a sequential ET–PT mechanism, wherein inner-sphere ET initially localizes charge on CO_2_, enabling a feasible barrier for PT to yield *COOH. This framework clarifies how solvent electrostriction impedes the initial ET rate, while simultaneously accelerating protonation through ion–dipole stabilization of the anionic intermediate.

### 3.2 Comparison of ET–PT and PCET kinetics

**Fig. 2:**
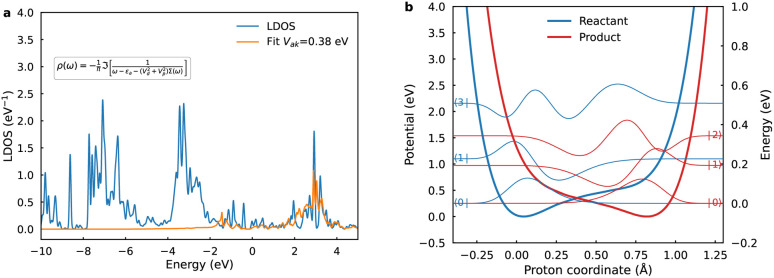
Electronic coupling and proton potentials at the crossing seam. (a) Electronic coupling matrix element *V*_*ak*_ obtained from the Newns–Anderson Hamiltonian fit at the centroid distance of 2.42 Å along the *z*-axis between the CO_2_ molecule and the surface Cu atoms. (b) Proton potentials and corresponding vibrational wavefunctions for the contributing vibrational state for the reactant and product state at the equilibrium donor–acceptor distance *R* of 2.70 Å. The energy scale on the left corresponds to the proton potential, while the scale on the right corresponds to the vibrational energy levels of the proton.

To elucidate kinetic distinctions between sequential ET–PT and concerted PCET pathways, we calculated the electrochemical ET and concerted PCET rate constants. Inner-sphere ET entails electron injection from Cu d-band states into the CO_2_ lowest unoccupied molecular orbitals, *i.e.* 2p*. Electronic coupling at the diabatic crossing point was explicitly evaluated using the Newns–Anderson Hamiltonian (Fig. 2a), with the resulting values exceeding 0.38 eV. This contrasts with the lower-bound estimate of 0.11 eV obtained from a simplified Lorentzian fit (Fig. S11). This substantial coupling is consistent with strong chemisorption interactions. This large coupling indicates that the reaction proceeds on a single, ground-state adiabatic potential energy surface. In this regime, the reactant and the electrode share the transferring electron, the adiabatic TST is the appropriate framework for calculating the ET rate constant. While prior studies invoked Fermi's golden rule for ET rates *in vacuo*, solvent reorganization energy (*λ*) emerges as paramount at electrochemical interfaces.^22^ Structurally, forming CO_2_^−^ entails a linear-to-bent geometric reconfiguration, with charge states eliciting differential solvent polarization.

Solvent reorganization energy for inner-sphere ET was quantified *via* dual MLP-driven molecular dynamics trajectories on neutral and anionic diabatic surfaces. Physisorbed (*Q*_R_) and chemisorbed (*Q*_P_) geometries are chosen as the initial configurations on the neutral and anionic surfaces, equilibrated for 10 ns with snapshots every 1 ps (10 000 frames each). The probability distributions of the vertical energy gap for each diabatic state, as shown in Fig. S9 and S10. The computed *λ* of 1.46 eV reflects substantial energetic costs from interfacial water reorganization, driven by ion-dipole interactions-key to charge stabilization. Marcus theory then estimates the crossing barrier as 0.47 eV from *λ* and reaction free energy. However, for the subsequent calculation of the ET rate constant, the free-energy barrier of 0.37 eV obtained directly from the WT-MetaD simulation was employed, as it provides a more complete and dynamical description of the reaction pathway. Extending this to PCET,^43^ reactant geometries (proton on H_2_O) and product geometries (proton on *CO_2_^−^) were optimized with fixed other nuclei to yield *λ*_R_ = 1.76 eV and *λ*_P_ = 1.28 eV, respectively; the averaged *λ* ≈ 1.52 eV approximates the WT-MetaD reaction free energy of 0.27 eV. At the potential of zero charge (PZC, −0.5 V *vs.* SHE), *k*_ET_ ≈ 10^6^ s^−1^-markedly subdued relative to vacuum estimates of 10^15^ s^−1^.^22^ This disparity underscores the dominance of solvent reorganization, demonstrating that inner-sphere ET at metal/water interfaces cannot be captured by purely electronic descriptions but instead requires explicit treatment of bath-induced fluctuations and dissipative dynamics.

**Fig. 3:**
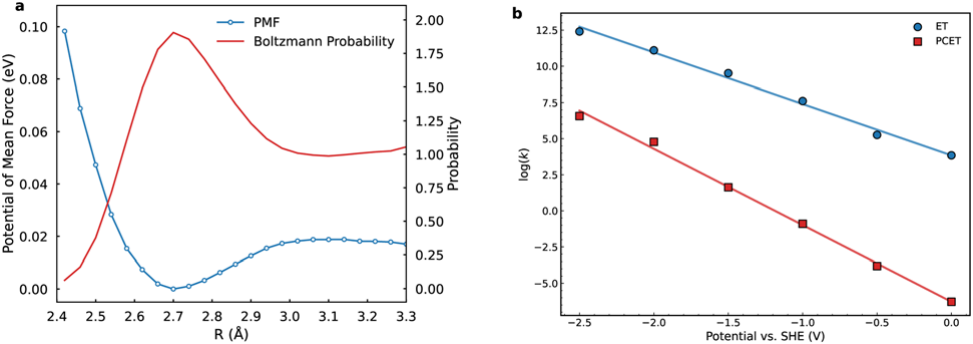
Thermodynamic and kinetic for PCET. (a) Potential of mean force (PMF) along the proton donor–acceptor distance R (blue line) and the associated normalized Boltzmann probability distribution (red line). (b) Potential-dependent rate constants for inner-sphere ET (blue) and concerted PCET (red) obtained from Landau–Zener rate theory.

Within the quantum-mechanical framework of PCET,^18,44^ the electron and proton are treated quantum mechanically, whereas the remaining nuclear coordinates are treated classically. The pivotal quantity dictating nonadiabatic PCET is the vibronic coupling *V*_*aµkν*_, which, along with the proton vibrational wavefunctions, exhibits pronounced dependence on the proton donor–acceptor distance *R*. For each fixed *R*, one-dimensional diabatic proton potentials and associated vibrational wavefunctions are computed along the trajectory linking equilibrium proton positions in reactant and product states. The equilibrium *R* was ascertained *via* the potential of mean force (PMF) along this coordinate using umbrella sampling, yielding the normalized Boltzmann probability distribution (Fig. 3a) with an equilibrium value of 2.70 Å. It should be noted that the PMF increases along the distance *R*, but does not grow unbounded, thus, the Boltzmann factor continues to contribute a non-negligible probability across the sampled coordinate range. Subsequently, one-dimensional diabatic proton potentials were constructed at discrete values of *R* ranging from 2.50 to 3.10 Å in 0.1 Å increments. For each fixed *R*, molecular dynamics trajectories of 1 ns duration were propagated on the adiabatic ML-PES, employing the SHAKE algorithm to constrain *R*. Averaged structures obtained from these trajectories served as diabatic crossing-point geometries for subsequent CDFT calculations, providing a set of one-dimensional proton potentials connecting reactant and product equilibrium proton positions.^45^

Fig. 2b illustrates diabatic proton potentials for both reduced and oxidized *CO_2_ states at *R* = 2.70 Å, additional potentials and wavefunctions are provided in the Fig. S12. The proton potentials are notably asymmetric; the reduced state potential minimum is located near the oxygen atom of CO_2_, whereas the oxidized state potential minimum is closer to the oxygen atom of H_2_O. Analysis indicates that decreasing *R* distances enhance proton vibrational wavefunction delocalization, substantially increasing overlaps between reactant and product ground-state wavefunctions. Conversely, at larger *R* distances, excited vibrational states progressively dominate rate constant contributions due to increased overlaps with ground product states.^46^

To accurately quantify the PCET rate constant, the individual *R*-dependent rate constants are integrated across the sampled range, each weighted by the Boltzmann probability distribution (Fig. 3a). At the equilibrium distance of 2.70 Å, significant contributions arise predominantly from transitions between the third excited reactant state and ground product state and from the first excited reactant and second excited product states. These excited-state transitions have substantial proton delocalization facilitating efficient vibronic coupling. Conversely, at a shorter distance of 2.50 Å, the primary contribution arises from ground-state transitions. Thus, rates at each *R* are governed by vibrational overlaps, favoring compact *R*; however, overall rates are modulated by sampling probabilities, biasing toward equilibrium *R*.^47^ This interplay underscores the multifaceted balance shaping PCET kinetics.

Potential-dependent rate constants were evaluated using adiabatic TST for ET and the Landau–Zener rate expression for concerted PCET. The electrostatic potential at the reaction plane, relative to PZC was modeled *via* a reported Gouy–Chapman–Stern (GCS) approach (Fig. S13), incorporating distance-dependent dielectric permittivity in the outer Helmholtz layer to accurately represent interfacial dielectric saturation effects.^40^ As illustrated in Fig. 3b, ET rate constants consistently exceed those of PCET across the examined potential range. This difference arises primarily from the inherently higher reorganization energy and diminished proton vibrational wavefunction overlaps intrinsic to PCET mechanisms. Kinetics are predominantly governed by ground-state vibrational transitions, capitalizing on substantial overlaps to enable efficient proton tunneling. Beyond the equilibrium *R*, excited vibronic states contribute approximately 25% to the PCET rate at the PZC when integrated over *R* distributions (Fig. S14). This mechanistic transition reflects a subtle balance among vibrational state populations, vibronic overlaps, and thermodynamic considerations. At shorter distances, excited states remain energetically prohibitive, leaving ground-state transitions driven by strong vibrational overlaps as the dominant contribution. At longer distances, despite the decreasing thermal accessibility and reduced conformational probability, excited vibronic states become increasingly favorable due to smaller free-energy gaps with the product state and substantial overlaps resulting from their vibrational delocalization.

Notably, the vibronic coupling strength initially increases with distance from 2.50 Å, driven by reduced vibrational energy level spacing and significant ground-state overlap integrals *S*_00_. Beyond the equilibrium distance of 2.70 Å, the coupling strength diminishes, as contributions are increasingly dominated by delocalized excited vibronic states whose Boltzmann populations *w*_*µ*_ decline with increasing vibrational energy. Nevertheless, these excited-state transitions remain essential, contributing significantly (∼25%) to the overall PCET rate due to their enhanced thermodynamic favorability at larger distances. Thus, the fractional contribution of each vibrational state to the PCET rate emerges from a complex interplay between vibrational overlaps *S*_*µν*_, the Boltzmann populations *w*_*µ*_, and the state-specific free energy gaps Δ*G*_*µ*0_.^45^

### 3.3 Solvent dynamics

**Fig. 4:**
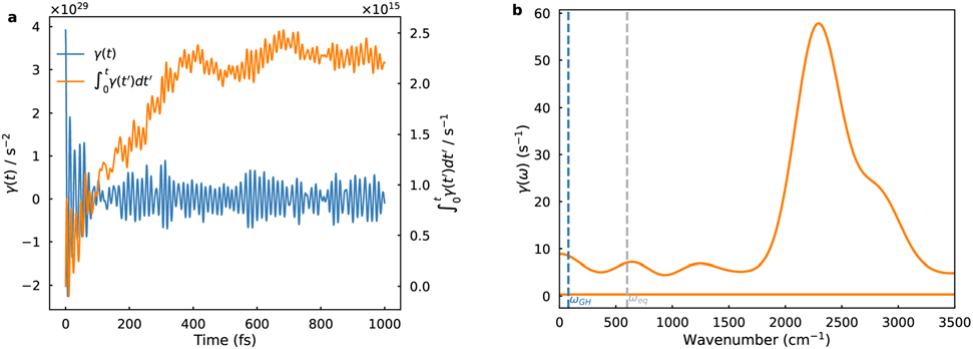
Solvent dynamics at the PCET crossing seam. (a) Time-dependent friction kernel, governing the solvent reorganization along the reaction coordinate. (b) Corresponding friction spectrum, revealing dominant interfacial vibrational modes that couple to the reaction coordinate.

The utilization of equilibrium distribution functions *W*_*µν*_(*R*) and *M*(*Ẋ*) in PCET theory for computing crossing-point sampling probabilities presupposes rapid solvent dynamics, wherein the solvent attains thermal equilibrium on a timescale much shorter than the reaction. This limit corresponds to the spatial diffusion regime of Kramers theory with Markovian friction. Nevertheless, polar solvent dynamics exert profound influence on reaction kinetics, particularly in processes entailing substantial charge redistribution at barrier crossings. Although curve-crossing formulas yield cusp-shaped barriers, solvent reorganization often emerges as the rate-determining factor. Furthermore, the Landau–Zener treatment presumes a constant velocity through the diabatic crossing; any velocity relaxation enhances adiabatic character and weakens nonadiabatic suppression. To mitigate solvent response delays stemming from inertial polarization, we adopt GH theory within the generalized Langevin equation (GLE) formalism, which embeds the reaction coordinate in a bath characterized by a frequency-dependent friction kernel. This framework is extended to PCET on diabatic FESs, with the friction kernel extracted from crossing point force autocorrelation functions (FACFs) computed from MLMD simulations, as depicted in Fig. 4. The friction kernel decays on a sub-100 fs timescale but exhibits pronounced oscillations, evidencing strong coupling between the reaction coordinate and interfacial solvent modes. The corresponding friction spectrum is dominated by a single band near 2500 cm^−1^, attributable to the O–H stretching manifold of water. These ultrafast vibrations intermittently modulate the diabatic gap and shape the stochastic sampling of the crossing seam. The high-frequency intramolecular vibrations concurrently govern two critical aspects: they dictate the equilibrium sampling of the classical coordinate *R* by modulating the hydrogen-bond network, and they serve as the primary bath responsible for dissipating energy and imposing Markovian friction on the reaction dynamics. Incorporating the GH correction thus allows the approach to go beyond the instantaneous equilibrium (Markovian) assumption and reveals how inertial polarization dynamics dampen the overall charge transfer rate.

## 4 Conclusions

We have developed a comprehensive quantum-classical multiscale framework to elucidate ET and PT kinetics in electrochemical CO_2_RR at the Cu(100)/H_2_O interface, integrating CDFT, MLMD, and a diabatic vibronic rate theory augmented by GH corrections within the GLE formalism. The major conclusions are summarized as follows:

(1) Distinct neural network potentials for the adiabatic ground state and for two charge-localized diabatic states are well trained, allowing efficient configurational sampling while preserving quantum-mechanical fidelity for electronic energies and forces.

(2) Our calculations unambiguously discriminate between sequential ET–PT and concerted PCET pathways, showing that the sequential ET–PT pathway outpaces the concerted PCET pathway by approximately 5 orders of magnitude at PZC.

(3) Frequency-resolved friction analysis identifies interfacial O–H stretching modes as the principal bath coordinates that modulate electronic-nuclear coupling and the Landau–Zener transition probability.

(4) Vibrationally excited reactant states contribute 25% of the total PCET rate, highlighting the kinetic importance of excited-state vibronic channels in electrochemical environments.^48^

(5) Acceleration of the rate-determining ET and PT events at the metal/aqueous interfaces can be achieved by stabilizing the *CO_2_^−^ intermediate, by engineering hydrogen-bond networks that transiently shorten donor–acceptor separations, and by tailoring interfacial dielectric response to reduce solvent-induced friction.

## Author contributions

Yun Yang: conceptualization, methodology, software, validation, formal analysis, investigation, data curation, writing – original draft, visualization. Gang Fu: conceptualization, resources, writing – review & editing, supervision, project administration, funding acquisition.

## Conflicts of interest

There are no conflicts to declare.

## Supplementary Material

SC-017-D5SC07385E-s001

## Data Availability

The data supporting the findings of this article are included in the supplementary information (SI). Supplementary Information: detailed information on the training errors of the MLPs, the CDFT setup, the electrical double layer model, the extrapolation of the vibronic coupling, friction kernel, and a comparison between the Newns–Anderson Hamiltonian and Lorentzian fitting for the electronic coupling matrix element, as well as additional computational details. See DOI: https://doi.org/10.1039/d5sc07385e.
