# Supplementary material for: Resolving sequential electron–proton transfer kinetics for electrochemical CO2 reduction at the Cu(100)/H2O interface via a quantum-classical framework
Source: Chem Sci. 2026 Feb 2;17(13):6419–25. doi: 10.1039/d5sc07385e (PMC12875385; doi:10.1039/d5sc07385e)
Supplement: SC-017-D5SC07385E-s001 [file SC-017-D5SC07385E-s001.pdf]

Supplementary Information (SI) for Chemical Science.
This journal is © The Royal Society of Chemistry 2026

Supporting Information

Resolving Sequential Electron-Proton Transfer Kinetics for Electrochemical CO_2 Reduction at the Cu(100)/H_2O Interface via a Quantum-Classical Framework

Yun Yang and Gang Fu*

College of Chemistry and Chemical Engineering, Xiamen University, Xiamen, China

E-mail: gfu@xmu.edu.cn

Phone: +86 13625012808. Fax: 86-592-2183047

Computational Methods

Electronic Structure Calculations

In this work, all density functional theory (DFT) calculations were performed using the CP2K/Quickstep package.[1] The DFT implemented in CP2K is based on a hybrid Gaussian plane wave (GPW) scheme. The orbitals were described by an atom centered Gaussian-type basis set, and an auxiliary plane wave basis set was used to re-expand the electron density in the reciprocal space. The Perdew-Burke-Ernzerhof (PBE) functional with Grimme's dispersion correction with Becke-Johnson damping (PBE-D3BJ) and the double-ζ plus polarization (DZVP) basis set were used.[2–4] An energy cutoff of 800 Ry was set for the plane-wave basis set. The core electrons were modeled using Goedecker-Teter-Hutter (GTH) norm-conserving pseudopotentials.[5,6] Periodic boundary conditions were applied in all dimensions. The wave function optimization was performed using matrix diagonalization method at Γ point. The convergence criterion for wave function optimization was set by a maximum electronic gradient of 1×10^{-7} a.u. and an energy difference tolerance between self-consistent field (SCF) cycles of 1×10^{-14} a.u., respectively. Constrained DFT calculations employed the outer SCF loop with a convergence threshold of 1×10^{-7} a.u.. Specially, the proton potentials for the seven different O-O distances began with the seven average structures. For each average structure, the hydrogen was moved along a grid of 36 points spanning the axis, and a single point CDFT calculation was performed using the triple-ζ valence double-polarized (TZV2P) basis set and a stricter convergence criterion of 1×10^{-8} a.u.. These energies were then interpolated to create a grid of 1024 points using a cubic spline interpolation scheme, as implemented in previously reported methodology.[7,8] Ab initio molecular dynamics (AIMD) simulations were performed in the canonical (NVT) ensemble using a Nosé-Hoover thermostat at 330 K, and integrated with a 0.5 fs timestep.

Workflow for Machine Learning Potentials Construction

Three separate machine learning potentials (MLPs) were constructed using a concurrent learning strategy combine with well-tempered MetaDynamics (WT-MetaD). The workflow contains three main components: training, exploration and labeling. These three steps are repeated iteratively until the final MLPs are accurate enough for atomic simulation which are implemented in ai2-kit package.[9] The initial structures for AIMD were prepared using the freely available PACKMOL and pre-equilibrated with 1 ns via classical NVT molecular dynamics using the SPC/E water potential.[10,11] Subsequently, 5 ps of CDFT-based AIMD simulations were carried out in the NVT ensemble, starting from both chemisorption and physisorption configurations. For each AIMD trajectory, 25 configurations were randomly sampled. This procedure generated six trajectories, from which potential energies and atomic forces were extracted to compose the initial training datasets.

In the training step, a committee of four MLPs was trained based on the energies and forces using the Deep Potential Smooth Edition (DeepPot-SE) descriptor with a cutoff radius of 6 Å.[12–14] The number of neurons in each hidden layer of the embedding net and the fitting net was [25, 50, 100] and [240, 240, 240], respectively. The MLPs were trained in 2×10^5 steps, with each step using a batch size of 1. During learning, the learning rate was reduced exponentially from 1×10^{-3} to 1×10^{-8}, where the learning rate was updated every 2000 steps. Both energies and atomic forces were included in the loss function for model optimization. The weight for energies in the loss function started from 0.02 and ended at 1, while that for atomic forces started from 1000 and ended at 1. In the exploration step, new configurations were generated by performing MD simulations based on one of the MLPs trained in the previous step. In each exploration step, MD simulations with various initial configurations were performed at various temperatures. Considering that MLPs are expected to be good at interpolating but poor at extrapolating, the deviations in the results predicted by the four MLPs trained in the previous step can be used to estimate in how far a given configuration is already represented by the existing training dataset. Configurations for which the deviations

in atomic forces laid between 0.2 and 0.4 eV/Å were selected as candidates to form additional training data. In each iteration, a certain number of these candidate configurations was chosen randomly from the candidate structures and added to the training dataset. In the labeling step, DFT calculations were performed for the selected configurations from the exploration step with the computational setup specified above. Through this iterative process, the final training datasets for the adiabatic, diabatic 1, and diabatic 2 potential energy surfaces comprised 4302, 3102, and 5440 configurations, respectively.

Free Energy Calculations

These workflows combined WT-MetaD with MLMD to obtain free energy profiles for CO_2 adsorption and proton-coupled electron transfer (PCET) step at different temperatures. As a collective variable, the distance of geometric centroid distance between the CO_2 molecule and the surface Cu atom along the z-axis was used for inner-sphere ET, for *CO_2-to-*COOH, two collective variables R_1 and R_2 were used, which is illustrated in Figure S1. To accelerate sampling along the free-energy surface, WT-MetaD was employed at 330 K, utilizing a Gaussian width of 0.2, a hill height of 5 kJ/mol, and a bias factor of 16.8. Minimum energy paths (MEP) were computed by optimizing the two-dimensional free-energy surfaces (FESs) using the Nudged Elastic Band (NEB) method, with the limited-memory Broyden-Fletcher-Goldfarb-Shanno (L-BFGS) algorithm serving as the optimizer via SciPy's implementation. The MD simulations were performed using LAMMPS and PLUMED in the NVT ensemble using a Nosé-Hoover thermostat at 330 K, and integrated with a 0.5 fs timestep.[15,16] WT-MetaD introduces a time-dependent bias Gaussian potential along the collective variable, enabling enhanced sampling of rare events in molecular simulations. By biasing the system away from previously sampled regions of configuration space, WT-MetaD allows the reconstruction of the FES as a function of the chosen collective variable. Each simulation was carried out for 10–20 ns to ensure convergence. To determine the equilibrium distribution and corresponding Boltzmann probability distribution for the distance

R between the CO_2 oxygen (proton acceptor) and H_2O oxygen (proton donor), umbrella sampling simulations were performed. A series of initial configurations was generated, each corresponding to a specific donor-acceptor distance within the range of 2.50 to 3.10 Å, with increments of 0.1 Å. In each configuration, the R was harmonically restrained at its target distance using an umbrella biasing potential of $200\,\mathrm{kJ/(mol \cdot \AA^2)}$. This restraint enabled efficient sampling of the configurational space within defined windows along the donor-acceptor reaction coordinate. Adjacent windows were spaced to ensure sufficient overlap of the sampled reaction coordinate, a prerequisite for robust potential of mean force (PMF) reconstruction. Molecular dynamics (MD) simulations were conducted for each window using the Nosé-Hoover thermostat (T = 330 K) and an integration timestep of 0.5 fs, as previously described. Each trajectory was propagated for 5 ns. Following simulation, the weighted histogram analysis method (WHAM) was employed to combine the sampled distributions from all windows. This analysis yielded the one-dimensional PMF along the reaction coordinate and the corresponding equilibrium Boltzmann probability distribution for the donor-acceptor distance.

The solvent reorganization free energy for the inner-sphere ET step, two independent molecular dynamics simulations with MLPs trained on the two diabatic surfaces are performed. Physisorbed (Q_R) and chemisorbed (Q_P) geometries were used as the initial configurations on the neutral and anionic surfaces, respectively; each system was equilibrated for 10 ns, and snapshots were stored every 1 ps, giving 10000 frames per trajectory. For every configuration Q, we computed the vertical energy gap as

$$\Delta E_R(Q) = \langle E_P(Q) - E_R(Q) \rangle_R, \quad \Delta E_P(Q) = \langle E_P(Q) - E_R(Q) \rangle_P \tag{1}$$

the angle brackets denote averages over the corresponding trajectory, thereby obtaining the energy gap variances δ_R^2 and δ_P^2 from the R- and P-trajectories.

$$\delta_R^2 = \langle (\Delta E_R - \langle \Delta E_R \rangle_R)^2 \rangle_R, \quad \delta_P^2 = \langle (\Delta E_P - \langle \Delta E_P \rangle_P)^2 \rangle_P \tag{2}$$

With the framework of Marcus theory and leveraging the linear-response approximation validated by free energy perturbation (FEP), the ET free energy difference (ΔF) and solvent contribution to the reorganization free energy are given by

$$\Delta F = -k_B T \ln \left\langle e^{-\beta \Delta E} \right\rangle_Q \tag{3}$$

$$\lambda_R = \frac{\delta_R^2}{2k_B T}, \quad \lambda_P = \frac{\delta_P^2}{2k_B T} \tag{4}$$

The distribution of ΔE for both states are Gaussian, as shown in Figure S8 and S9, confirming the validity of the linear-response approximation in Marcus theory for the solvent. This computational scheme was extended to quantify the reorganization energy for PCET. Here, the reactant state equilibrium geometry Q_R has the proton on its donor (H_2O), while the product state geometry Q_P has the proton on its acceptor (CO_2^-). To calculate $E_R(Q_P)$, the proton was optimized on the donor with all other nuclei fixed to Q_P. Conversely, for $E_P(Q_R)$, the proton was optimized on the acceptor with all other nuclei fixed to Q_R. These geometry optimizations were performed using the adiabatic machine learning potential function with the BFGS algorithm, as implemented in CP2K. The convergence criterion for these optimizations was set to a maximum force of 1×10^{-4} a.u..

Vibronic Coupling Calculations

For the inner-sphere ET, electron from the d-band of Cu transfer to the LUMO of CO_2. This charge injection is quantitatively described by the Newns-Anderson Hamiltonian:

$$H = \sum_k \epsilon_k c_k^\dagger c_k + \epsilon_a c_a^\dagger c_a + \sum_k \left[V_{ak} c_a^\dagger c_k + V_{ak}^* c_k^\dagger c_a \right] \tag{5}$$

where ϵ_k are the energies of the metal one-electron states within the conduction band, ϵ_a is the LUMO energy level, and V_{ak} quantifies the metal-adsorbate coupling strength. The

resulting adsorbate local density of states (LDOS) derives from the retarded Green's function:

$$\rho_a(\epsilon) = -\frac{1}{\pi}\,\mathrm{Im}\left[\frac{1}{\epsilon - \epsilon_a - \Sigma(\epsilon) + i\delta}\right] \tag{6}$$

with the hybridization self-energy $\Sigma(\epsilon)$:

$$\Sigma(\epsilon) = \mathcal{P}\int \frac{\Lambda(\epsilon')}{\epsilon - \epsilon'}\mathrm{d}\epsilon' - i\pi\Delta(\epsilon) \tag{7}$$

where $\Delta(\epsilon) = \pi \sum_k |V_{ak}|^2 \delta(\epsilon - \epsilon_k)$ is the d-band projection coupling function, Λ denotes the shift of the orbital energy caused by this interaction. We than applied this methodology to extract the electronic coupling matrix element at the crossing point. Two hundred transition configurations were sampled from the MD trajectory at 1 ps intervals, with the ensemble-averaged electronic coupling obtained by averaging the computed values.

Within the theoretical framework of PCET,[17–19] the center quantity that governs nonadiabatic PCET is the vibronic coupling $V_{a\mu k\nu}$. The vibronic coupling and proton vibrational wavefunctions depend strongly on the proton donor-accept distance R. To construct the relevant potential energy surfaces, diabatic proton potentials were generated by performing CDFT calculations at averaged reactant and product geometries for discrete values. These geometries served as crossing-point structures, yielding a family of one-dimensional diabatic proton potentials connecting the equilibrium proton positions in the reactant and product states. The one-dimensional Schrödinger equation was solved numerically for each potential to obtain the proton vibrational eigenfunctions and their overlap integrals.

Solvent Friction Kernel

The adiabatic transition state theory and diabatic vibronic representation of PCET theory relies critically on the assumption of fast solvent dynamics.[18,20] This assumption posits that solvent relaxation occurs on a timescale significantly shorter than the reaction itself, ensuring that solvent degrees of freedom maintain thermal equilibrium throughout the reaction process.

Moreover, Landau-Zener theory requires constant velocity near the crossing seam, velocity relaxation otherwise facilitate adiabatic processes and diminishing nonadiabatic suppression. To address solvent response lags arising from inertial polarization effects, we incorporate the Grote-Hynes (GH) theory which based on the generalized Langevin equation (GLE), explicitly describes the dynamical coupling between the solvent environment and the reactive system via a frequency-dependent friction kernel.[21,22] According to the GH theory, reactive frequency is determined by solving the GH equation:[23,24]

$$\omega_{GH}^2 - \omega_{\text{eq}}^2 + \omega_{GH} \int_0^\infty \gamma_{\text{TS}}(t) e^{-\omega_{GH} t} \text{d}t = 0 \tag{8}$$

Here, ω_{GH} and ω_{eq} denote the reactive frequency and equilibrium frequency, respectively. The equilibrium frequency $\approx 600cm^{-1}$ was obtained from normal mode analysis using DFT. The time-dependent friction kernel $\gamma(t)$ can be calculated from the autocorrelation of the forces exerted on the reaction coordinate.

$$\gamma(t) = \frac{\langle F(0)F(t) \rangle}{mk_B T} \tag{9}$$

Where m is the reduced mass of the reaction coordinate. To understand in more detail how the environment dynamics influence the reaction we need to identify which motions couple with the reaction coordinate and how they contribute to the friction kernel. With this purpose we have obtained the friction spectra as the Fourier transforms of the friction kernels:

$$\gamma(\omega) = \int_{-\infty}^{+\infty} \gamma(t) e^{i\omega t} \text{d}t \tag{10}$$

Although curve-crossing models yield cusp-shaped barriers where solvent dynamics minimally barrier crossing, solvent reorganization often emerges as the rate-determining factor. To characterize this solvent-reaction coordinate coupling, we compute the friction kernel through constrained simulations initiated at the PCET crossing point using the RATTLE algorithm.

For each configuration, 200 trajectories were propagated on diabatic potential energy surfaces (PESs). The force autocorrelation functions (FACFs) were then calculated from the forces on the reaction coordinate. The friction coefficient, which quantifies the dissipative coupling between the reaction coordinate and the solvent environment, was obtained by integrating the time-dependent friction kernel over time. All MD simulations were performed at 330 K with a time step of 0.1 fs to ensure a good convergence of the constraint under the NVT ensemble.

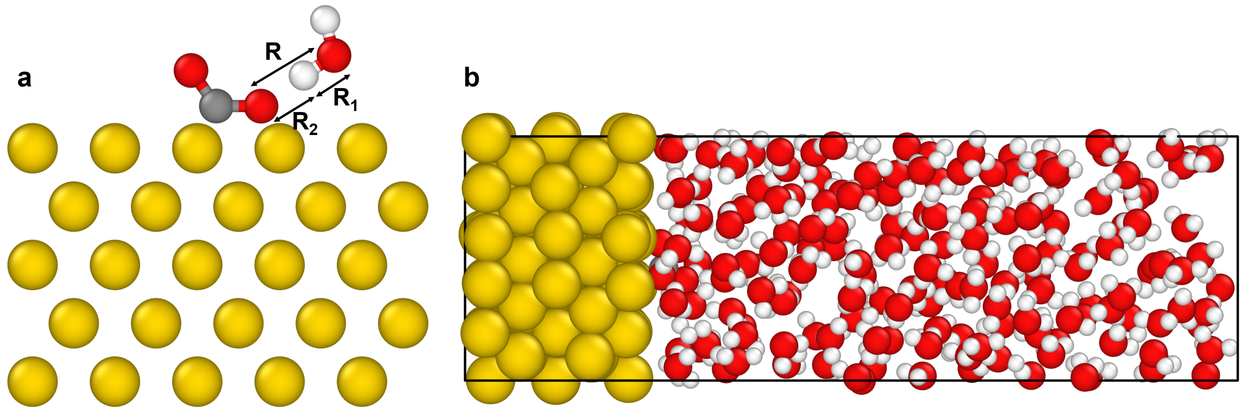

Figure S1. Illustration of atomic models used. (a) Collective variables used for well-tempered metadynamics and umbrella sampling. (b) 3D periodic supercell used in molecular dynamics simulations.

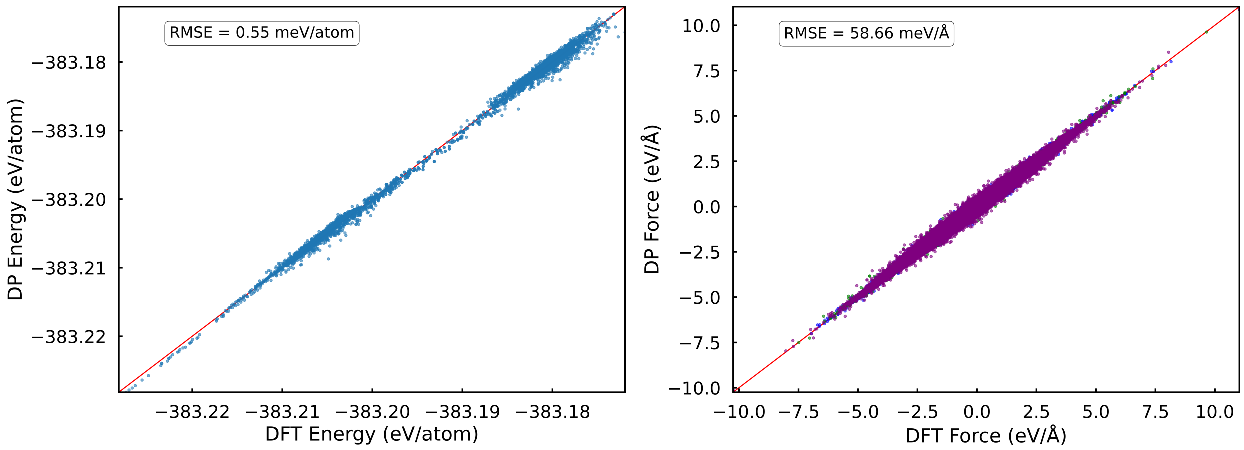

Figure S2. Prediction errors of the MLP for adiabatic state potential energies and atomic forces on training datasets.

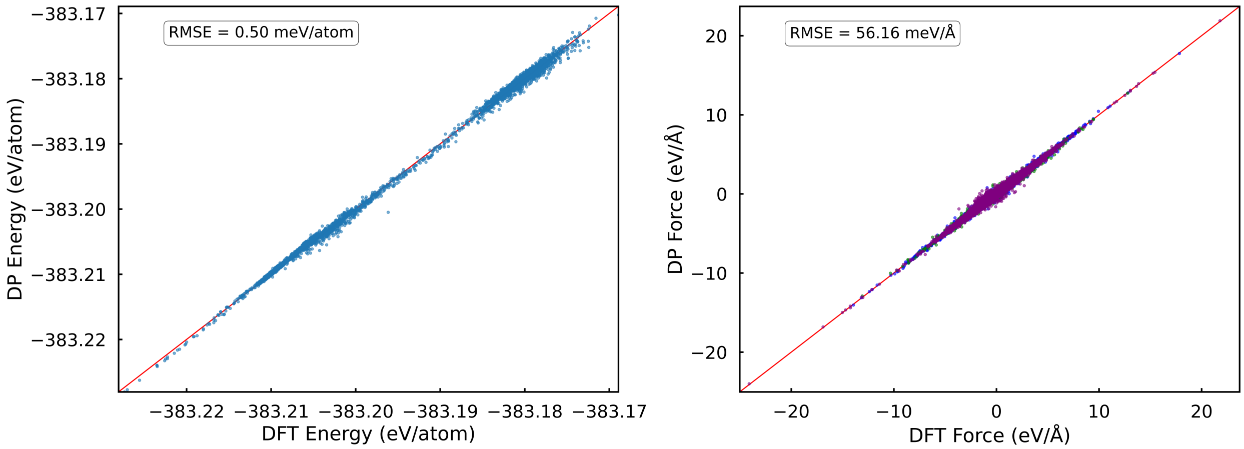

Figure S3. Prediction errors of the MLP for neutral state potential energies and atomic forces on training datasets.

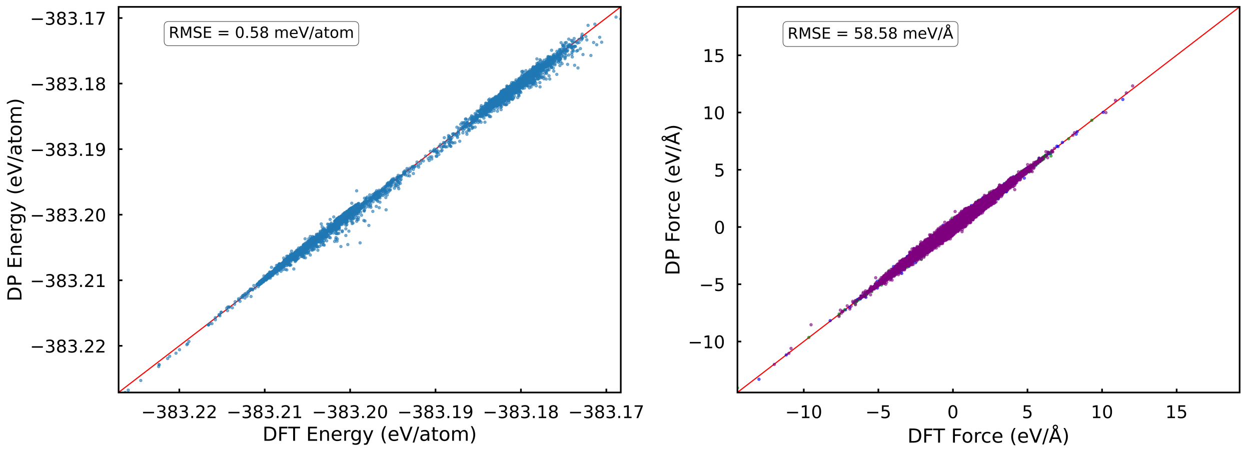

Figure S4. Prediction errors of the MLP for anionic state potential energies and atomic forces on training datasets.

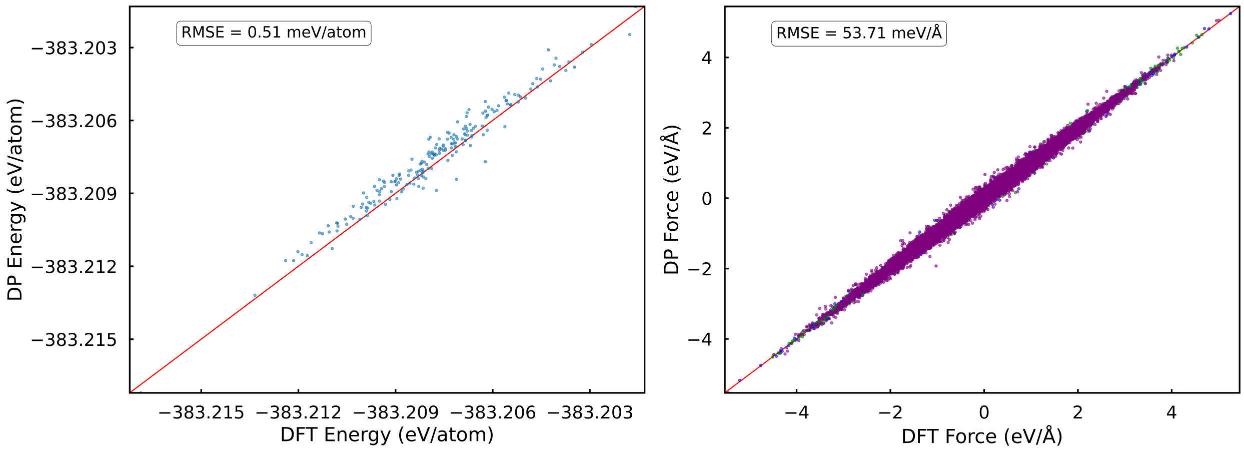

Figure S5. Validation errors in MLP-predicted adiabatic state potential energies and atomic forces using datasets sampled from WT-MetaD trajectories.

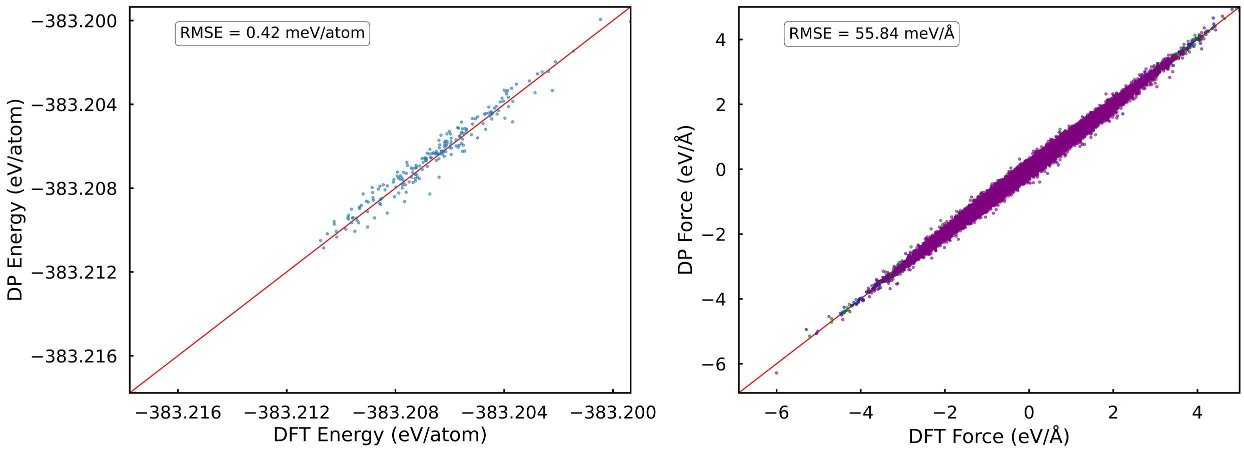

Figure S6. Validation errors in MLP-predicted neutral state potential energies and atomic forces using datasets sampled from WT-MetaD trajectories.

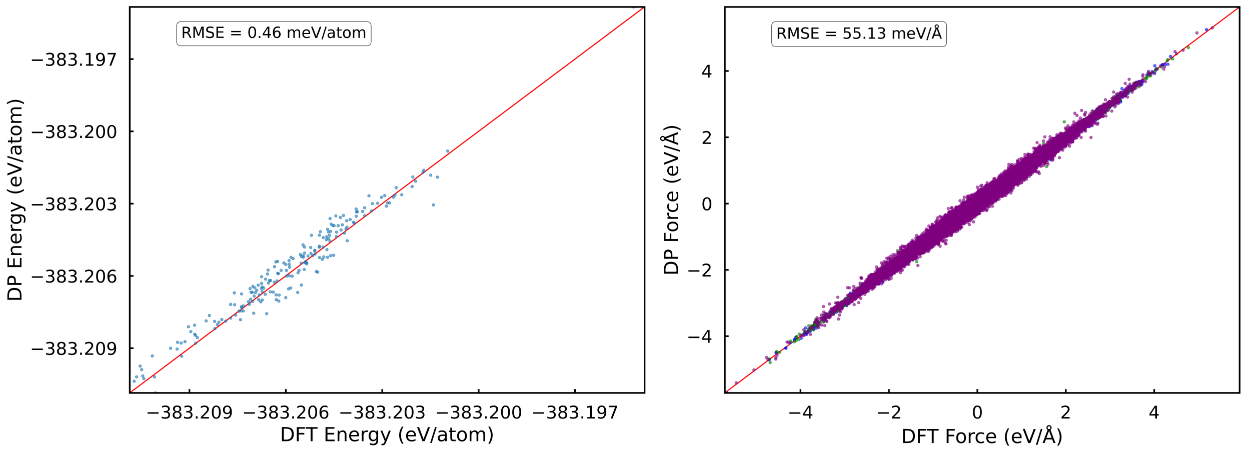

Figure S7. Validation errors in MLP-predicted anionic state potential energies and atomic forces using datasets sampled from WT-MetaD trajectories.

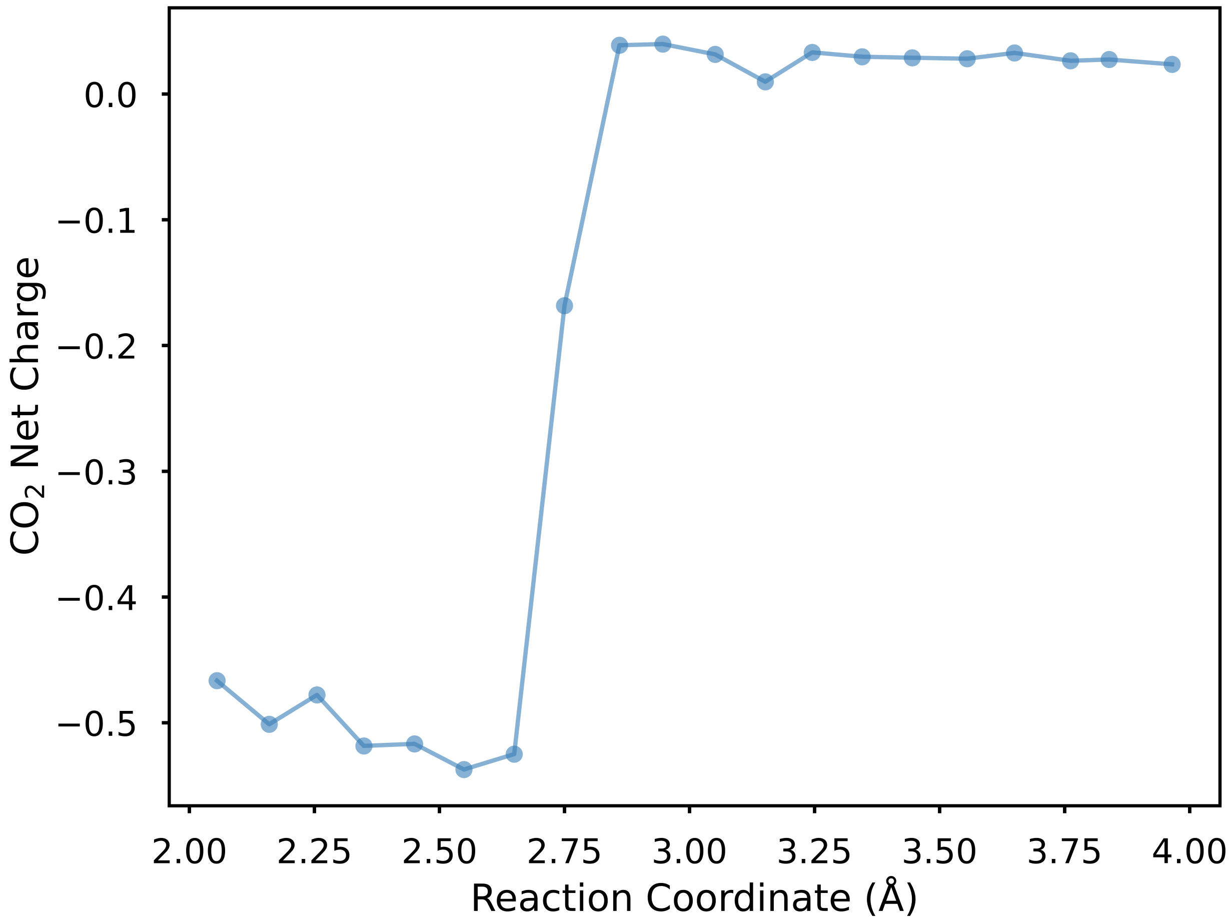

Figure S8. Evolution of the mean net Mulliken charge on CO_2 along the reaction coordinate for adiabatic ET. The data is extracted from 20 configurations per window sampled across 20 windows of the WT-MetaD trajectory.

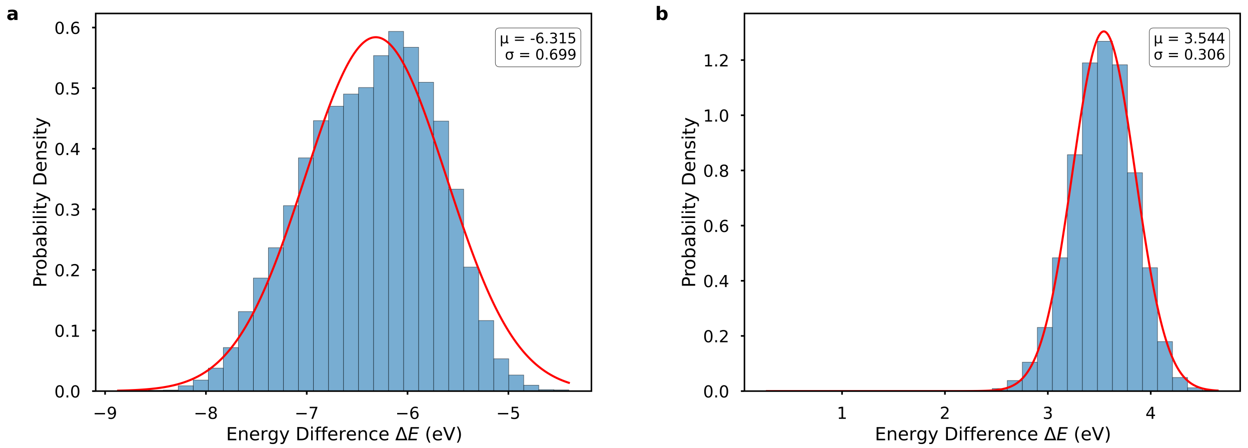

Figure S9. Probability distributions of the energy gap (a) $\Delta E_R(Q)$ for the reactant state and (b) $\Delta E_P(Q)$ for the product state along the inner-sphere ET.

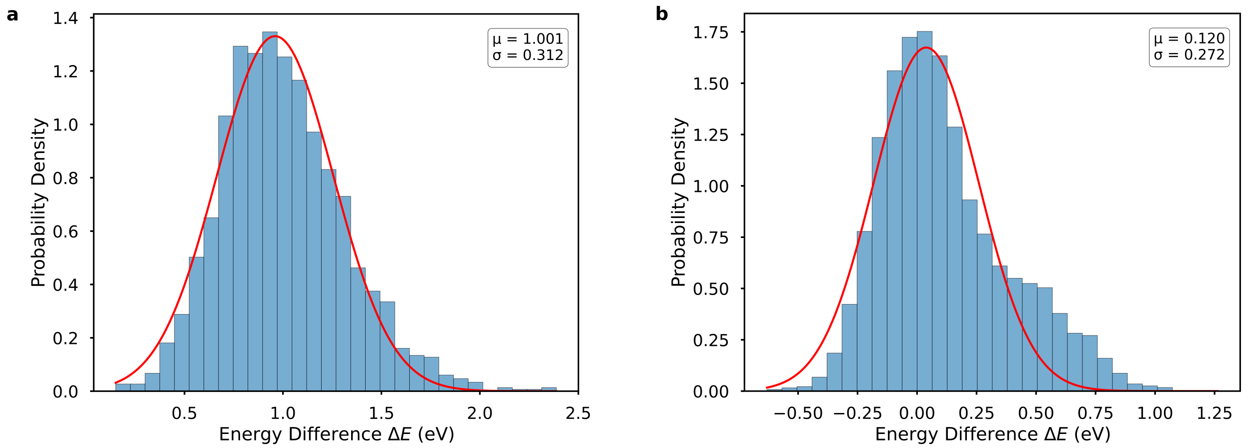

Figure S10. Probability distributions of the energy gap (a) $\Delta E_R(Q)$ for the reactant state and (b) $\Delta E_P(Q)$ for the product state along the CEPT.

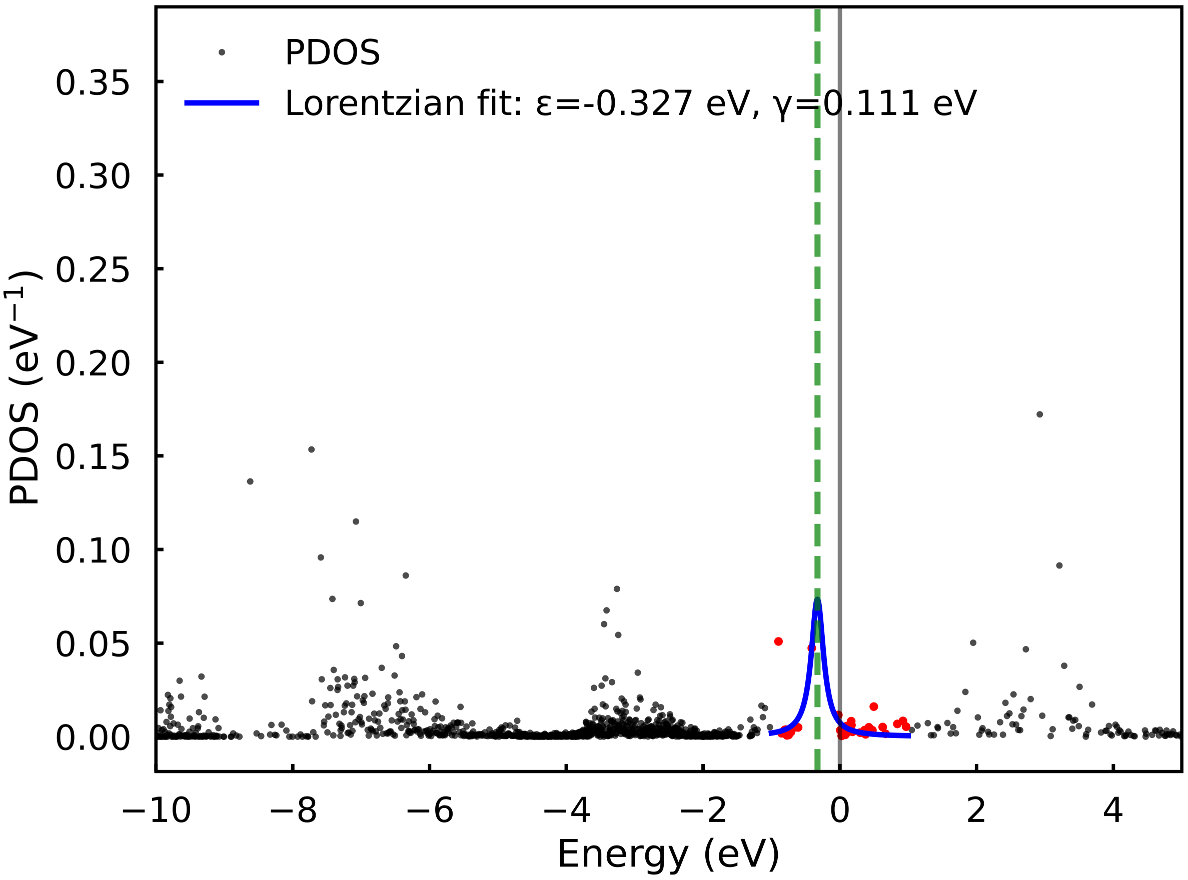

Figure S11. Lorentzian fit (blue line) to the chemisorption function at the Γ-point. The resonance energy $\epsilon = -0.327$ eV, with a half width at half maximum $\gamma = 0.111$ eV.

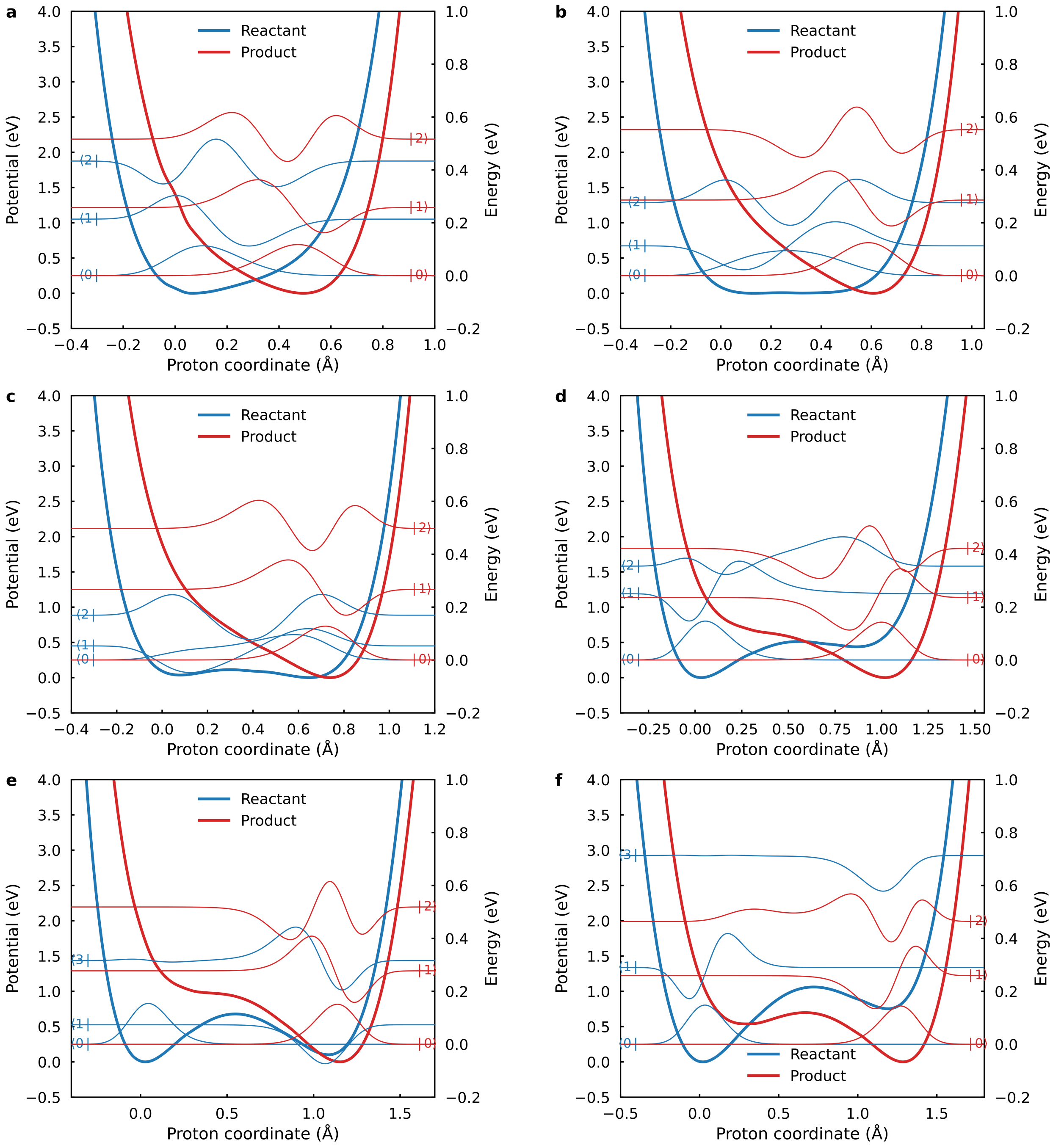

Figure S12. Proton potentials and corresponding vibrational wavefunctions at donor-acceptor distance of (a) 2.5 (b) 2.6 (c) 2.8 (d) 2.9 (e) 3.0 and (f) 3.1 Å. The energy scale on the left corresponds to the proton potential, while the scale on the right corresponds to the vibrational energy levels of the proton.

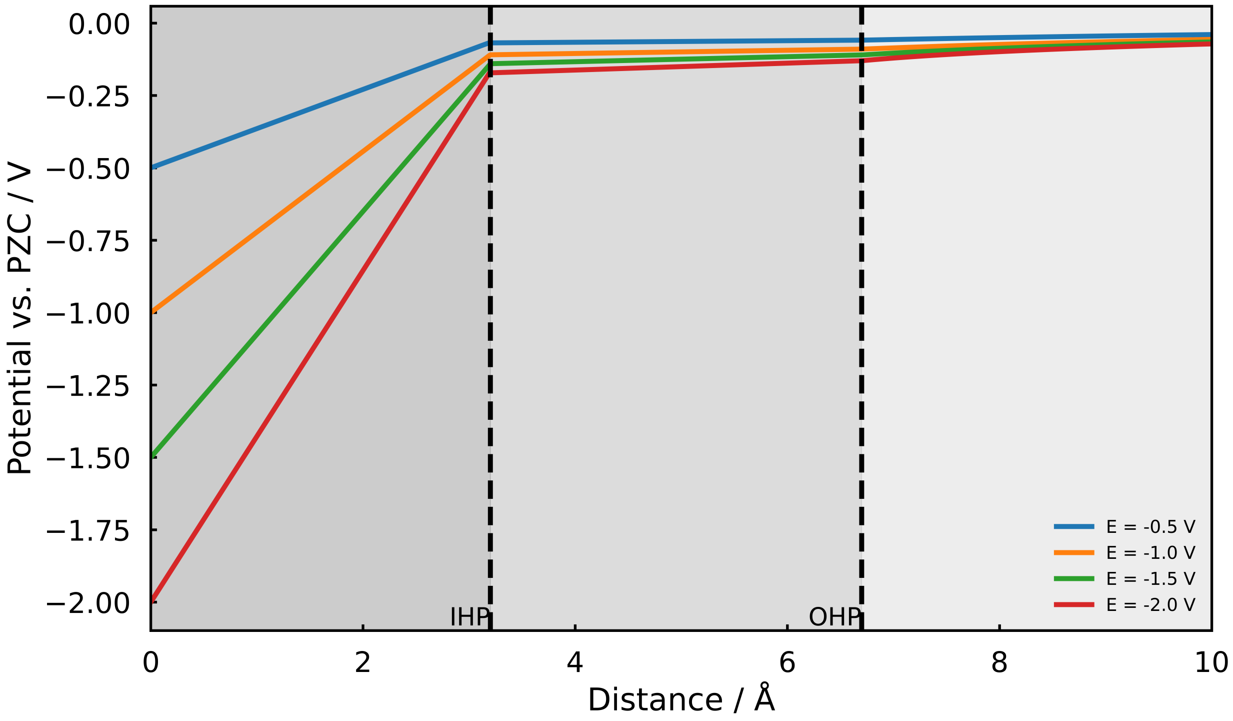

Figure S13. The electrostatic potential as a function of distance from the electrode for various applied potentials relative to the potential of zero charge.

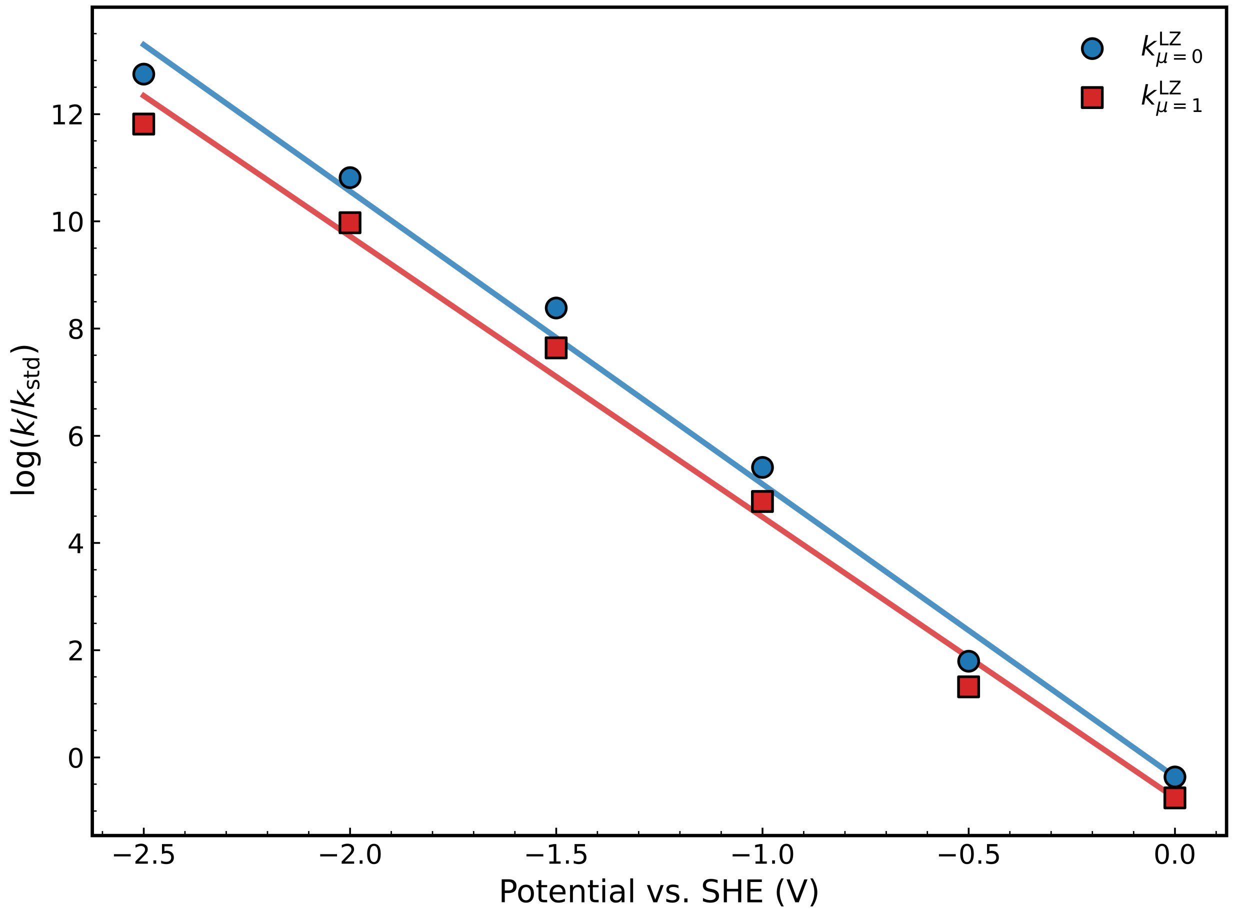

Figure S14. Potential-dependent rate constants for concerted PCET processes originating from the ground vibrational state and the first vibrationally excited state.

References

(1) Kühne, T. D. et al. CP2K: An electronic structure and molecular dynamics software package - Quickstep: Efficient and accurate electronic structure calculations. *J. Chem. Phys.* **2020**, *152*, 194103–194150.

(2) Perdew, J. P.; Burke, K.; Ernzerhof, M. Generalized gradient approximation made simple. *Phys. Rev. Lett.* **1996**, *77*, 3865–3868.

(3) Grimme, S.; Antony, J.; Ehrlich, S.; Krieg, H. A consistent and accurate ab initio parametrization of density functional dispersion correction (DFT-D) for the 94 elements H-Pu. *J. Chem. Phys.* **2010**, *132*, 154104–154123.

(4) VandeVondele, J.; Hutter, J. Gaussian basis sets for accurate calculations on molecular systems in gas and condensed phases. *J. Chem. Phys.* **2007**, *127*, 114105–114114.

(5) Hartwigsen, C.; Goedecker, S.; Hutter, J. Relativistic separable dual-space Gaussian pseudopotentials from H to Rn. *Phys. Rev. B* **1998**, *58*, 3641–3662.

(6) Goedecker, S.; Teter, M.; Hutter, J. Separable dual-space Gaussian pseudopotentials. *Phys. Rev. B* **1996**, *54*, 1703–1710.

(7) Horvath, S.; Fernandez, L. E.; Soudackov, A. V.; Hammes-Schiffer, S. Insights into proton-coupled electron transfer mechanisms of electrocatalytic H2 oxidation and production. *Proc. Natl. Acad. Sci. U.S.A.* **2012**, *109*, 15663–15668.

(8) Warburton, R. E.; Soudackov, A. V.; Hammes-Schiffer, S. Interfacial Proton-Coupled Electron Transfer via Localized Trap States on Metal Oxide Surfaces. *J. Phys. Chem. C* **2024**, *128*, 7903–7912.

(9) Guo, Y.-X.; Zhuang, Y.-B.; Shi, J.; Cheng, J. ChecMatE: A workflow package to automatically generate machine learning potentials and phase diagrams for semiconductor alloys. *J. Chem. Phys.* **2023**, *159*, 094801–094813.

(10) Martinez, L.; Andrade, R.; Birgin, E. G.; Martinez, J. M. PACKMOL : A package for building initial configurations for molecular dynamics simulations. *J. Comput. Chem.* **2009**, *30*, 2157–2164.

(11) Berendsen, H. J. C.; Grigera, J. R.; Straatsma, T. P. The missing term in effective pair potentials. *J. Phys. Chem.* **1987**, *91*, 6269–6271.

(12) Wang, H.; Zhang, L.; Han, J.; E, W. DeePMD-kit: A deep learning package for many-body potential energy representation and molecular dynamics. *Comput. Phys. Commun.* **2018**, *228*, 178–184.

(13) Zeng, J. et al. DeePMD-kit v2: A software package for deep potential models. *J. Chem. Phys.* **2023**, *159*, 054801–054825.

(14) Zeng, J. et al. DeePMD-kit v3: A Multiple-Backend Framework for Machine Learning Potentials. *J. Chem. Theory Comput.* **2025**, *21*, 4375–4385.

(15) Thompson, A. P.; Aktulga, H. M.; Berger, R.; Bolintineanu, D. S.; Brown, W. M.; Crozier, P. S.; in 't Veld, P. J.; Kohlmeyer, A.; Moore, S. G.; Nguyen, T. D.; Shan, R.; Stevens, M. J.; Tranchida, J.; Trott, C.; Plimpton, S. J. LAMMPS - a flexible simulation tool for particle-based materials modeling at the atomic, meso, and continuum scales. *Comput. Phys. Commun.* **2022**, *271*, 108171–108205.

(16) Tribello, G. A.; Bonomi, M.; Branduardi, D.; Camilloni, C.; Bussi, G. PLUMED 2: New feathers for an old bird. *Comput. Phys. Commun.* **2014**, *185*, 604–613.

(17) Venkataraman, C.; Soudackov, A. V.; Hammes-Schiffer, S. Theoretical formulation of nonadiabatic electrochemical proton-coupled electron transfer at metal-solution interfaces. *J. Phys. Chem. C* **2008**, *112*, 12386–12397.

(18) Lam, Y. C.; Soudackov, A. V.; Hammes-Schiffer, S. Theory of electrochemical proton-coupled electron transfer in diabatic vibronic representation: Application to proton

discharge on metal electrodes in alkaline solution. *J. Phys. Chem. C* **2020**, *124*, 27309–27322.

(19) Hutchison, P.; Cui, K.; Zhong, J.; Hammes-Schiffer, S. Tutorial on computing nonadiabatic proton-coupled electron transfer rate constants. *J. Chem. Phys.* **2025**, *163*, 091501–091516.

(20) Lam, Y. C.; Soudackov, A. V.; Hammes-Schiffer, S. Kinetics of Proton Discharge on Metal Electrodes: Effects of Vibrational Nonadiabaticity and Solvent Dynamics. *J. Phys. Chem. Lett.* **2019**, *10*, 5312–5317.

(21) Northrup, S. H.; Hynes, J. T. The stable states picture of chemical reactions. I. Formulation for rate constants and initial condition effects. *J. Chem. Phys.* **1980**, *73*, 2700–2714.

(22) Grote, R. F.; Hynes, J. T. The stable states picture of chemical reactions. II. Rate constants for condensed and gas phase reaction models. *J. Chem. Phys.* **1980**, *73*, 2715–2732.

(23) Roca, M.; Moliner, V.; Tuñón, I.; Hynes, J. T. Coupling between protein and reaction dynamics in enzymatic processes: Application of Grote-Hynes theory to catechol O-methyltransferase. *J. Am. Chem. Soc.* **2006**, *128*, 6186–6193.

(24) Kanaan, N.; Roca, M.; Tun, I.; Mart, S.; Moliner, V. Application of Grote-Hynes theory to the reaction catalyzed by thymidylate synthase. *J. Phys. Chem. B* **2010**, *114*, 13593–13600.
